# From neuroleptic malignant syndrome to malignant catatonia: The impact of ciprofloxacin in a chronic psychiatric patient

**DOI:** 10.1192/j.eurpsy.2025.1242

**Published:** 2025-08-26

**Authors:** M. Lo Monaco, A. Cuautle Bastida, A. Sabater Gil

**Affiliations:** 1 Psychiatry; 2 Internal Medicine, Parc Sanitari Sant Joan de Déu, Barcelona, Spain

## Abstract

**Introduction:**

We present the case of a 58-year-old male patient with a history of bipolar disorder, schizoaffective disorder, renal insufficiency and diabetes insipidus who developed malignant catatonia (MC), possibly related to the administration of ciprofloxacin. In a first admission to our care facility, the patient presented a neuroleptic malignant syndrome (NMS) due to antipsychotic medication, with partial remission following the discontinuation of these drugs and introduction of benzodiazepine (BZD) and dantrolene treatment. However, after this first hospital discharge, the patient required a second hospitalization due to a worsening of his general condition, apparently related to a urinary tract infection (UTI). During this last admission, antibiotic therapy with ciprofloxacin was administered, resulting in the worsening of his neuropsychiatric condition and developing clinical symptoms compatible with catatonia. The clinical presentation of his condition eventually requires treatment with BZD and electroconvulsive therapy (ECT).

**Objectives:**

The aim of this work is to describe the course and therapeutic approach of a case of MC possibly induced by ciprofloxacin, clarifying the differential diagnosis between NMS and MC.

**Methods:**

A thorough review of the patient’s clinical history was performed from the onset of symptoms in the care facility through his ICU and inpatient hospitalizations. Pharmacological changes were documented, including antipsychotic interruption and BZD administration. Treatment with ECT was initiated after an insufficient response to BZD. Finally, a literature review was conducted to explore the relationship between MC and ciprofloxacin.

**Results:**

Initially, the patient showed a partial remission of the catatonic symptoms with BZD and dantrolene after discontinuing antipsychotics, achieving a temporary stabilization. However, after the introduction of ciprofloxacin, he developed a severe catatonic symptoms characterized by central fever, extreme rigidity, mutism, and withdrawal from the environment. Despite BZD treatment, the catatonic symptoms persisted, leading to the decision to initiate ECT. After multiple sessions, the patient experienced a significant improvement on autonomic, motor and cognitive symptoms of catatonia, as well as a more robust clinical stabilization.

**Conclusions:**

This case suggests a possible association between the administration of ciprofloxacin and the onset of MC in a chronic psychiatric patient with a history of NMS. The favorable response to ECT highlights the utility of this therapeutic option in the management of severe catatonia when other treatments are not fully effective. The chronology on this case suggests a neurotoxic effect of ciprofloxacin, emphasizing the importance of considering interactions between antibiotics and psychiatric pathologies in vulnerable patients.

**Disclosure of Interest:**

None Declared

